# Insight into functionality and bioactive properties of whey protein/beta-glucan complex discrepancy during different pH-induced phase transition

**DOI:** 10.1016/j.fochx.2026.104343

**Published:** 2026-09-01

**Authors:** Heyang Xu, Zhishen Mu, Han Wang, Xin Wen, Jialun Hu, Zhanmei Jiang, Munkh-Amgalan Gantumur

**Affiliations:** a Key Laboratory of Dairy Science (Northeast Agricultural University), Ministry of Education, College of Food Science, Northeast Agricultural University, Harbin 150030, China; b Inner Mongolia Enterprise Key Laboratory of Dairy Nutrition, Health & Safety, Inner Mongolia Mengniu Dairy (Group) Co., Ltd., Huhhot 011500, China; c College of Food Science and Engineering, Inner Mongolia Agricultural University, Hohhot 010018, China

**Keywords:** Phase region, Whey protein isolate, Oat beta glucan, Functionality, Bioactive properties

## Abstract

This study aimed to elucidate the differences in functional and bioactive properties of whey protein isolate(WPI)-oat beta-glucan(OBG) mixed system during pH-induced phase transition. Four representative phase regions were identified, including co-soluble region at pH 2.0 (CSRpH2.0), soluble complex region at pH5.5 (SCRpH5.5), and insoluble complex region at pH 4.5 (ISRpH4.5) and co-soluble region at pH 7.0 (CSRpH7.0). The WPI-OBG mixture (WG) in CSRpH2.0 had the highest content of irregular secondary structures compared to the other phase regions. WG in SCRpH5.5 possessed higher foaming and emulsifying properties than that in ISRpH4.5. Compared with other phase regions, WG in CSRpH2.0 exhibited superior in vitro bioactivity. The principal component analysis showed WG in CSRpH2.0 had the highest comprehensive scores compared with those in other phase regions. The elucidation of differences in functionality and bioactive properties among protein-polysaccharide complexes from different phase regions provide a key theoretical basis for their targeted application in food industry.

## Highlights

• There were significant discrepancies between WG in different phase regions.
• WG in CSRpH2.0 had highest loose structures than that in other phase regions.
• FA/FS and EAI/ESI of WG in SCRpH5.5 was higher than ISRpH4.5.
• WG in CSRpH2.0 showed the best functionality and bioactive properties.

## 1 Introduction

Non-covalent interactions between proteins and polysaccharides represent a promising strategy for constructing functional complexes with enhanced functional and bioactive properties. For example, foam stability of soy protein isolate-polysaccharides complex was higher than that of soy protein isolate (Yameng Han et al., 2024). Whey protein isolate (WPI)-tremella polysaccharide complexes exhibited significantly higher emulsifying activity index and emulsifying stability index compared with WPI at pH of 4.5 (Ma et al., 2025). The mechanical properties of anionic xanthan gum-camellia seed protein complex emulsion gels were notably higher than single camellia seed protein carried emulsion gels (Lin et al., 2025). The DPPH radical scavenging activity of dandelion polysaccharides-WPI complex was enhanced compared with dandelion polysaccharides (Yujun Han et al., 2024). The protein-polysaccharide complexes formed through non-covalent interactions have been extensively employed as emulsion stabilizer, fat substitutes, nano-encapsulation materials, and edible films in the food industry (Kong et al., 2022).

The non-covalent interactions between proteins and polysaccharides include electrostatic interactions, hydrogen bonds and hydrophobic interactions (Schmitt & Turgeon, 2011). The non-covalent interactions between proteins and polysaccharides are weak and susceptible to system pH, which influences ionization of functional groups in proteins and polysaccharides (Lan et al., 2020). As the pH of the system changes, the mixed protein-polysaccharide system shifts between three states: thermodynamic incompatibility of two phase, complex coacervation of one phase and co-solubility of one phase, causing the phase transition. For instance, Zheng et al. (2025) confirmed that WPI and strong polyelectrolyte dextran sulfate bind via electrostatic interactions, thereby causing a phase transition. Igartúa et al. (2022) reported that phase behavior of WPI-soluble soybean polysaccharides (SSPS) system depended on pH, WPI:SSPS ratio, and NaCl concentration. Moreover, the phase separation of type-A gelatin-dextran mixture was divided into four steps, including droplet coalescence, droplet migration, primary and stable phase separation through a static multiple light scattering technique (Q. Wang et al., 2022). Thus, knowledge and control of phase transitions in protein-polysaccharide systems are critical for developing foods with desirable texture, stability and sensory properties.

WPI accounts for approximately 20% of milk protein and primarily consists of β-lactoglobulin, α-lactalbumin and bovine serum albumin (Mangano et al., 2019). Due to its surface-active properties, high nutrition value, excellent biocompatibility, and low processing cost, WPI has been widely used in pH-induced protein-polysaccharide phase transition studies (Ladda et al., 2024; Li et al., 2024; Zheng et al., 2025). Oat beta glucan (OBG) is a linear polysaccharide with several hydroxyl groups present in oat kernels (Daou & Zhang, 2012). These structural features endow OBG with excellent physicochemical properties, such as high solubility (Rohilla et al., 2025), high viscosity (Anjali et al., 2025), and a suitable electrostatic charge (pKa = 2.0–2.5). Most importantly, distinct from many other soluble polysaccharides, OBG exhibits unique biological activities, including cholesterol reduction, diabetes prevention, and gut microbiota modulation (Valido et al., 2021).

Furthermore, our previous work (Hu et al., 2025b) also demonstrated the pH-induced phase transformation mechanism of the WPI-OBG mixed system. Nevertheless, their discrepancy in structure, functional and bioactive properties between different phase regions, including the co-soluble region (CSR), soluble complex region (SCR), and insoluble complex region (ISR), have not been elucidated. Hence, structure, foaming properties, emulsifying properties, DPPH free radical scavenging capacity, α-amylase inhibitory activity, and α-glucosidase inhibitory activity were evaluated to investigate the differences in WPI-OBG complexes among the three phase regions. This study would be beneficial for predicting and designing the protein-polysaccharide functional ingredients with targeted properties, such as excellent functionality or high bioactive properties.

## 2 Materials and methods

### 2.1 Materials

WPI (93.77%) was obtained from Mullins Whey Company, USA. OBG (93%, M_w_ = 2.098 × 10^5^ Da) was purchased from Shaanxi Benhe Bioengineering Co., China.

### 2.2 Construction of WPI-OBG aqueous system induced by different pH values

The WPI and OBG were weighed and dissolved in distilled water to prepare solutions with a concentration of 1.0 mg/mL, respectively. Then, the solutions were magnetically stirred at room temperature for 4 h to ensure complete dissolution. A 0.02% (*w*/*v*) sodium azide solution was added to the WPI or OBG solution to prevent the bacterial growth. Next, the WPI solution and OBG solution were blended at a ratio of 1 to 1. The WPI-OBG mixture was labeled as WG. Finally, the pH value of WG was adjusted to 2–7 using HCl and NaOH with varying concentrations to investigate the pH-induced phase behavior.

### 2.3 Turbidity

The turbidity of WPI, OBG, and WG samples exposed to different pH values was measured by UV spectrophotometer (UV-6100, Shimadzu Corporation, Japan) at 600 nm. The concentrations of WPI and WG were fixed at 1.0 mg/mL. The measurement was repeated three times at 25 °C.

### 2.4 Measurement of particle size and zeta-potential

The particle size of WPI and zeta-potential of WPI, OBG, and WG were measured using a particle size and zeta potential analyzer (NANO ZS90, Malvern, UK) (Li et al., 2026). Initially, sample solutions (2 mg/mL) were equilibrated at room temperature for 60 s before measurement. The particle size of OBG and WG was analyzed by laser particle size analyzer (Ji’ nan Weina Granule Instrument Co., Ltd., Shandong, China). The results were demonstrated as median particle size (D_50_), which reliably and objectively displayed the actual particle size of sample (Silva et al., 2013). The obscuration of the samples was maintained within the range of 10–20%. The refractive index of the samples was set to 1.520, and the absorption index was set to 0.1.

### 2.5 Fluorescence spectrum

The fluorescence spectrum of WPI, OBG, and WG were scanned by fluorescence spectrophotometer (F-7000, Hitachi High Technologies Corporation, Japan) at 298 K based on the method of W.-D. Wang et al. (2022), and the samples were diluted to 0.1 mg/mL before analysis. The excitation wavelength (λ_ex_) was 280 nm, emission wavelength (λ_em_) was 290–450 nm, excitation slit and emission slit was 2.5 nm. To minimize internal filtration effect, correction was conducted with the formula as follows (Huang et al., 2023):

$$F=F_{\text{Inⅈt}}\times 10^{\left(A_{\mathrm{ex}}+A_{\mathrm{em}}\right)∕2}$$

where F is fluorescence intensity after correction, F_In*i*t_ is fluorescence intensity without correction, A_ex_ and A_em_ are the absorbance of OBG at the excitation wavelength and emission wavelength, respectively.

### 2.6 Surface hydrophobicity

The surface hydrophobicity of WPI, OBG, and WG was acquired by fluorescence spectrophotometer following the method of (Yan et al., 2026). Prior to analysis, WPI and WG solutions were prepared at a series of concentrations ranging from 0.1 to 0.5 mg/mL with distilled water. A 50 μL aliquot of 8.0 mmol/L ANS solution was mixed with 3.0 mL of each sample solution. The surface hydrophobicity of each sample was the slope between its fluorescence intensity and corresponding concentration.

### 2.7 Three-dimensional fluorescence spectroscopy

The three-dimensional fluorescence spectra of WPI, OBG, and WG were obtained using a fluorescence spectrophotometer (F-7000, Hitachi High Technologies Corporation, Japan) according to the method of Xu et al. (2022). The sample concentration was adjusted to 0.1 mg/mL. The excitation wavelength ranged from 200 to 350 nm, and emission wavelength scanning range was set from 200 to 500 nm at 298 K.

### 2.8 Fourier transform infrared spectrometer (FTIR)

The FTIR spectra of WPI, OBG, and WG were determined at full wavelength (4000 cm^−1^-400 cm^−1^) using a Fourier transform infrared spectrometer (Nicolet iS50, Thermo Fisher, Madison, USA) (Huang et al., 2026a). The samples (5 mg/mL) were mixed with KBr. Subsequently, the mixtures were ground and pressed into thin films. The resolution was 4 cm^−1^ and 32 scans were performed. The Peak-fit 4.12 software was applied for the quantitative analysis of the secondary structure of the sample.

### 2.9 Transmission electron microscope (TEM) analysis

The morphology of WPI, OBG, and WG was observed using a TEM (Hitachi, Japan) according to the method described by Y. Jiang et al. (2021). Firstly, the sample solutions (0.1 mg/mL) were stained with uranyl acetate (1%, *w*/*v*). Then, the stained samples were placed onto 200-mesh carbon-coated copper grids to observe the size and morphology of the samples.

### 2.10 Foaming properties

The foaming properties of WPI, OBG, and WG were determined following the method of (Huang et al., 2026b)with appropriate modifications. Initially, 15 mL of 10 mg/mL sample was whipped using a high-speed disperser (IKA, Germany) at 10000 rpm for 2 min in 100 mL volumetric cylinder. The foam volume was detected at 0 and 30 min. The foaming properties were calculated using the following equations:

Foaming ability (FA) (%)=$\frac{V0}{V}$×100%.

Foam stability (FS) (%) =$\frac{V_{30}}{V_{0}}$×100%.where V is volume of sample solution, V_0_ and V_30_ are the volume of foam at 0 and 30 min.

### 2.11 Emulsifying properties

The emulsifying activity index (EAI) and emulsion stability index (ESI) of WPI, OBG, and WG were determined (Huang et al., 2024). A mixture of soybean oil (1 mL) and sample solution (3 mL, 5 mg/mL) was homogenized and emulsified by a high-speed disperser. SDS solution was added to the emulsion samples collected from the bottom layer at 0 and 10 min. The absorbance was measured at 500 nm. The emulsifying properties were calculated by the following formula:

$$\mathrm{EAI}\left(m^{2}/g\right)=\frac{2\times 2.303}{C\times \left(1-\varphi \right)\times 10}\times A_{500}\times N$$

$$\mathrm{ESI}\left(\%\right)=\frac{A_{10}}{A_{0}}\times 100\%$$

where A_500_ is the absorbance at 500 nm at 0 min, C is the concentration of protein (mg/mL), φ is the volume fraction of the oil phase in the emulsion (0.25), N is the dilution factor, A_0_ and A_10_ were the absorbance at 0 min and 10 min, respectively.

### 2.12 DPPH free radical scavenging capacity

The DPPH free radical scavenging capacity of WPI, OBG, and WG was obtained following method of Chang et al. (2007). The sample solution (40 μL, 5 mg/mL) was blended with DPPH solution prepared in ethanol (160 μL, 0.1 mmol/L). The mixtures were kept in the dark for 30 min, and the absorbance was measured at 517 nm. The DPPH free radical scavenging capacity was calculated using the following formula:

$$\text{DPPH free radical scavenging capacity}\left(\%\right)=\left(1-\frac{A-A_{i}}{A_{j}}\right)\times 100\%$$

where A is the absorbance of the sample mixed with DPPH ethanol solution, A_i_ is the absorbance of the sample mixed with ethanol, and A_j_ is the absorbance of DPPH ethanol solution.

### 2.13 α-amylase inhibitory activity

The α-amylase inhibitory activity of WPI, OBG, and WG was determined according to the method described by Griffiths (1981). The starch solution was mixed with 0.1 mol/L PBS buffer (pH 7.0) and heated in a water bath at 60 °C for 8 min. Then, α-amylase and sample solution (5 mg/mL) were blended with the above solution. After reacting for 5 min, NaOH and DNS solution were added. The absorbance of sample was detected at 660 nm. The α-amylase inhibitory activity was calculated by the following equation:

$$\alpha -\mathrm{amylase}\;\text{inhibitory activity}\left(\%\right)=\left(\frac{A_{\text{control}}-A_{\text{sample}}}{A_{\text{control}}}\right)\times 100\%$$

where A_control_ and A_sample_ are the absorbance of blank and sample, respectively.

### 2.14 α-glucosidase inhibitory activity

The α-glucosidase inhibitory activity of WPI, OBG, and WG was measured according to method of Jiang et al. (2021). The sample solution (50 μL, 5 mg/mL) was mixed with α-glucosidase (100 μL). The mixture solution was incubated at 37 °C for 10 min, followed by the addition of *p*-nitrophenyl-α-D-glucopyranoside and further incubation for 20 min. Then, sodium carbonate was added. The absorbance was determined at 405 nm. The α-glucosidase inhibitory activity was calculated as follows:

$$\alpha -\text{glucosidase inhibitory activity}\left(\%\right)=\left(\frac{A_{\text{control}}-A_{\text{sample}}}{A_{\text{control}}}\right)\times 100\%$$

where A_control_ and A_sample_ are the absorbance of blank and sample, respectively.

### 2.15 Statistical analysis

All experiments were repeated at least three times. SPSS 22.0 software (SPSS Inc., Chicago, IL, USA) was used for analysis of variance (ANOVA). Duncan test was used to analyze the significant differences of the average at *P* < 0.05. The experimental data were plotted using Origin 2021 software. The data was downscaled with Principal component analysis (PCA) according to the principle of eigenvalues greater than 1.

## 3 Results and discussion

### 3.1 pH-induced phase transition region

**Fig. 1:**
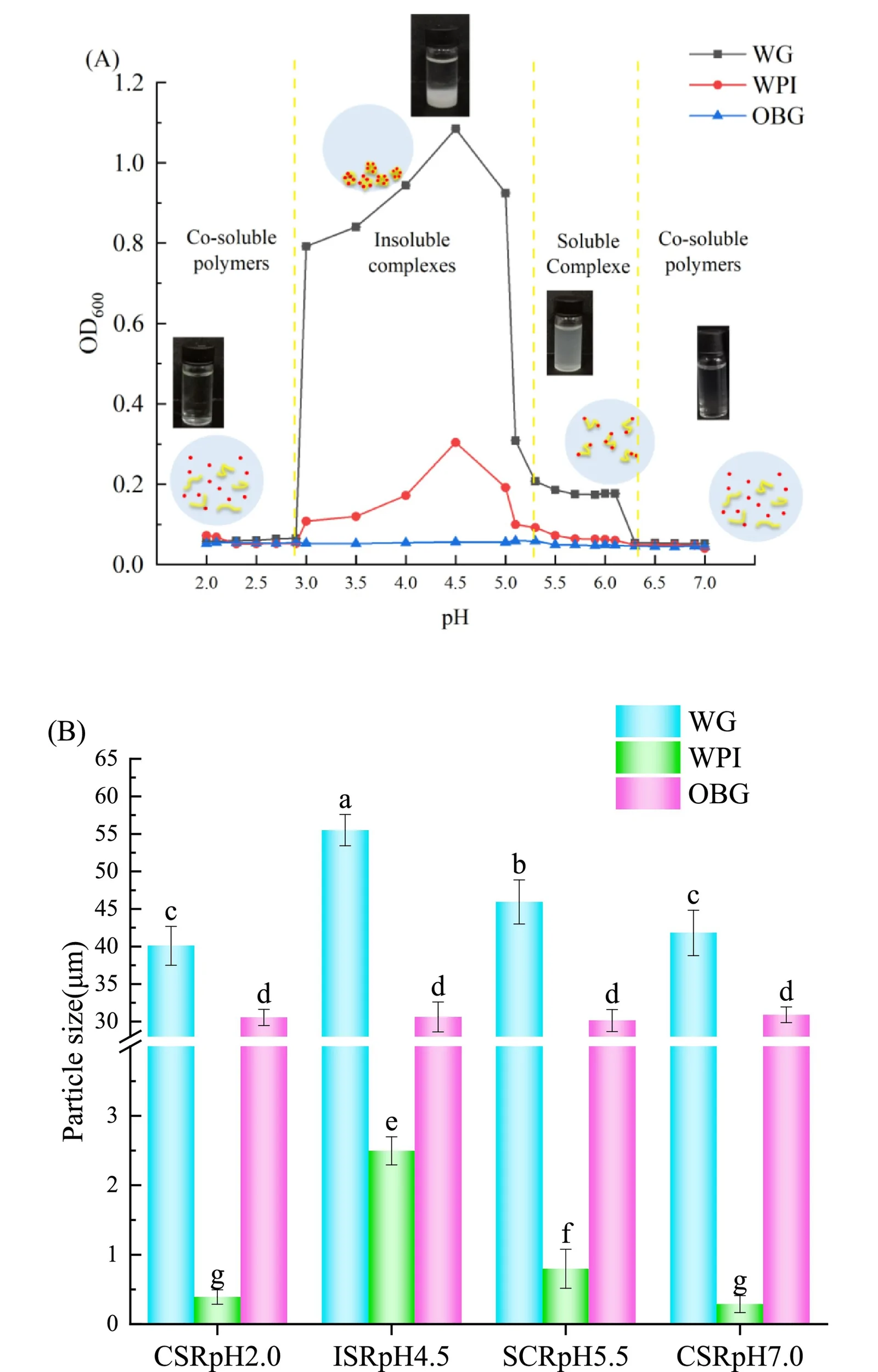

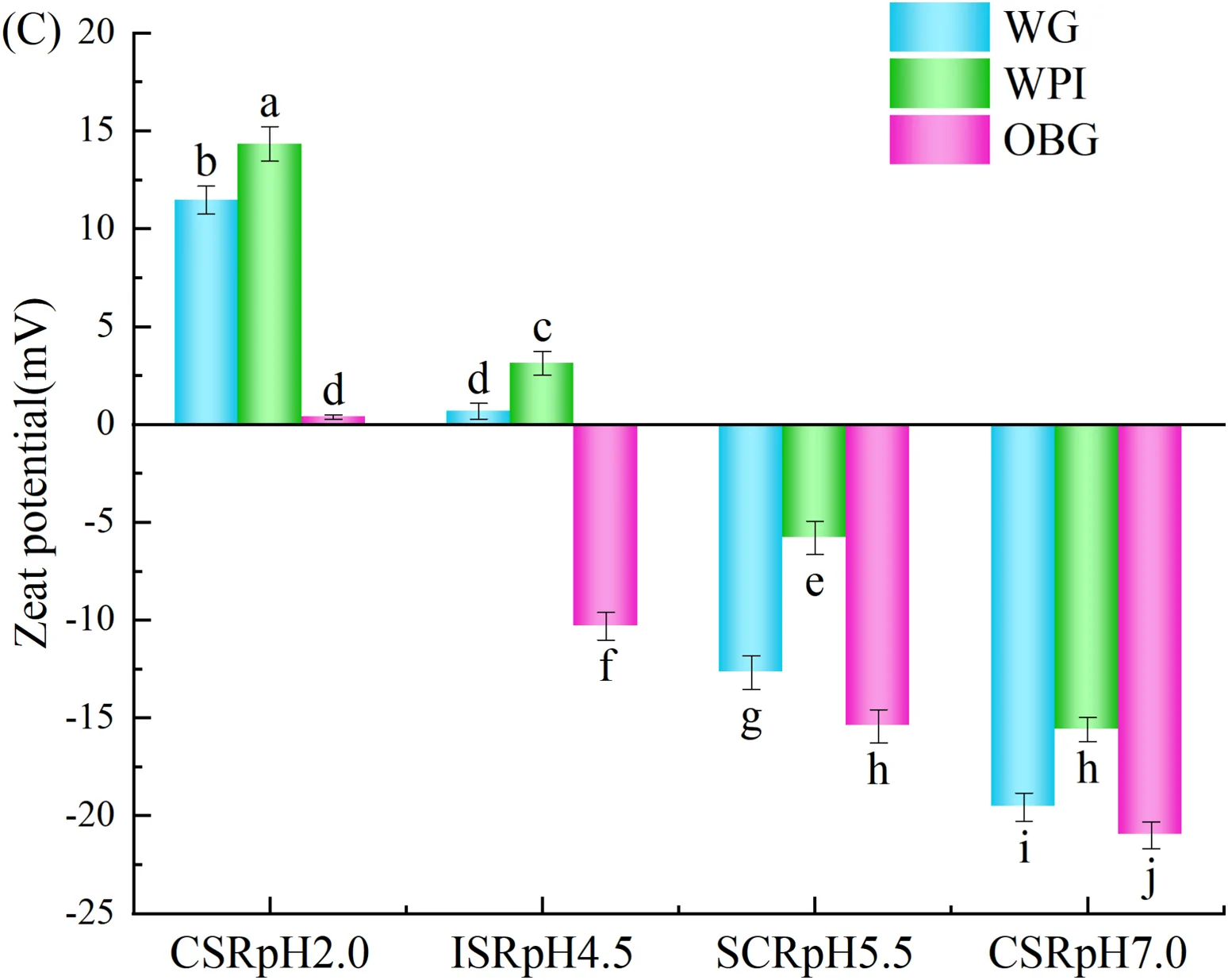
The turbidity(A), particle size D_50_ (B) and Zeta-potential (C) of WPI, OBG and WG at different phase regions. CSRpH2.0 is WPI-OBG co-soluble region at pH 2.0. ISRpH4.5 is WPI-OBG insoluble complex region at pH 4.5. SCRpH5.5 is WPI-OBG soluble complex region at pH 5.5. CSRpH7.0 is WPI-OBG co-solubility region at pH 7.0. Lowercase letters (a, b, c) indicate significant differences in different phase regions (*P* < 0.05). The same letters indicate no significant difference in different phase regions (*P* > 0.05).

The turbidity is commonly used to indicate the formation and dissociation of protein-polysaccharide complexes (Archut et al., 2023). As shown in Fig. 1A, the phase behavior of the WG system was mainly induced by changes in pH value. The WG system was transparent at pH values ranging from 6.3 to 7.0, and the similar negative charges of WPI and OBG (shown in Fig. 1C) generated electrostatic repulsion, thereby inhibiting the formation of WPI-OBG complexes. This suggested that the pH range from 6.3 to 7.0 corresponded to the co-soluble region (CSR). As the pH decreased to the range from 6.3 to 5.3, the turbidity of WG system was increased, indicating the formation of WPI-OBG complexes. No phase separation was observed in the WG system after 12 h of standing, indicating that that the formed complexes remained soluble and this pH range corresponded to the soluble complex region (SCR). Hadian et al. (2016) also reported that the soluble complex region of β-lactoglobulin-Persian gum mixture was generated at the pH around 5.30. With further acidification, the pH range from 2.9 to 5.3 showed a further increase in WG turbidity, which reached the maximum value at pH 4.5 (close to the isoelectric point (pI) of WPI), accompanied by phase separation, indicating formation of the insoluble complex region (ISR). A pervious study also found that the mixed system of soybean protein-soy soluble polysaccharide mixture exhibited phase separation at a pH close to the isoelectric point of soybean (Wu et al., 2022). As the pH decreased below 2.9, the turbidity of the mixed system declined, indicating gradual dissociation of the WPI-OBG complexes and a return to the co-soluble region (CSR). The co-soluble state of *β*-lactoglobulin–chitosan mixtures was also found under strongly acid or alkaline conditions (Mounsey et al., 2008). The WG at pH 2.0, 4.5, 5.5, and 7.0 was selected to represent the complex in the CSR at pH 2.0–2.9, ISR at pH 2.9–5.3, SCR at pH 5.3–6.3, and CSR at pH 6.3–7.0, respectively, with the aim of studying the differences in functional and bioactive properties between different phase regions.

### 3.2 Particle size and zeta-potential

The particle size (D_50_) and zeta-potential are important indictors for exploring the changes of protein-polysaccharide complex with pH (Zhao et al., 2023). Fig. 1B shows D_50_ of WG, WPI and OBG in different phase regions. WPI in ISRpH4.5 exhibited the highest D_50_, followed by SCRpH5.5, whereas CSRpH2.0 and CSRpH7.0 showed the lowest values. WPI was easily aggerated at pH values close to its pl (approximately 4.5), thus causing an increase in particle size. The high particle size was also found when the pH reached to the pI of chickpea protein isolate (Wang et al., 2022). No significant difference in D_50_ was observed for OBG among the four phase regions (*P* > 0.05). For WG, its D_50_ in ISRpH4.5 was the highest, followed by that in SCRpH5.5, whereas CSRpH2.0 and CSRpH7.0 showed the lowest values. This was related to the WPI-OBG interactions and aggregation degree of WPI-OBG complex.

Fig. 1C presents the zeta-potential of WG, WPI, and OBG in different phase regions. Both WPI and OBG showed different zeta-potential in CSRpH2.0, ISRpH4.5, SCRpH5.5, and CSRpH7.0, which might be related to the variation in pH among different phase regions. A similar change was also found in ovalbumin-propylene glycol alginate complex system in different pH (Zhang et al., 2023). In CSRpH2.0, the surface charged groups of OBG (pK_a_ = 2.0–2.5) became partially protonated under highly acidic conditions, which caused it to carry slight positive charges. The WPI (pI = 4.5–5.0) also carried positive charges at pH 2. Therefore, electrostatic repulsion occurred between WPI and OBG, preventing phase separation. In SCRpH5.5, WG had a lower absolute value of zeta-potential than that in CSRpH7.0 and higher than that in ISRpH4.5. This indicated that the electrostatic repulsion between WPI and OBG was weakened, allowing the formation of WPI-OBG complexes and masking their surface charges. The WPI-OBG complex was soluble and no aggregates were observed in WPI-OBG complex. In ISRpH4.5, WPI and OBG with opposite net charges formed WPI-OBG complexes through electrostatic attraction. The zeta-potential of WG was close to 0, aggregation of WPI-OBG complexes occurred, resulting in an insoluble state. It was also found that the surface charge of WPI-SSPS complex was offset by the electrostatic interactions, thus causing decrease of absolute value of zeta-potential (Igartúa et al., 2024). In CSRpH7.0, both WPI and OBG carried strong negative charges, and their electrostatic repulsion maintained WG in a co-soluble state.

**Fig. 2:**
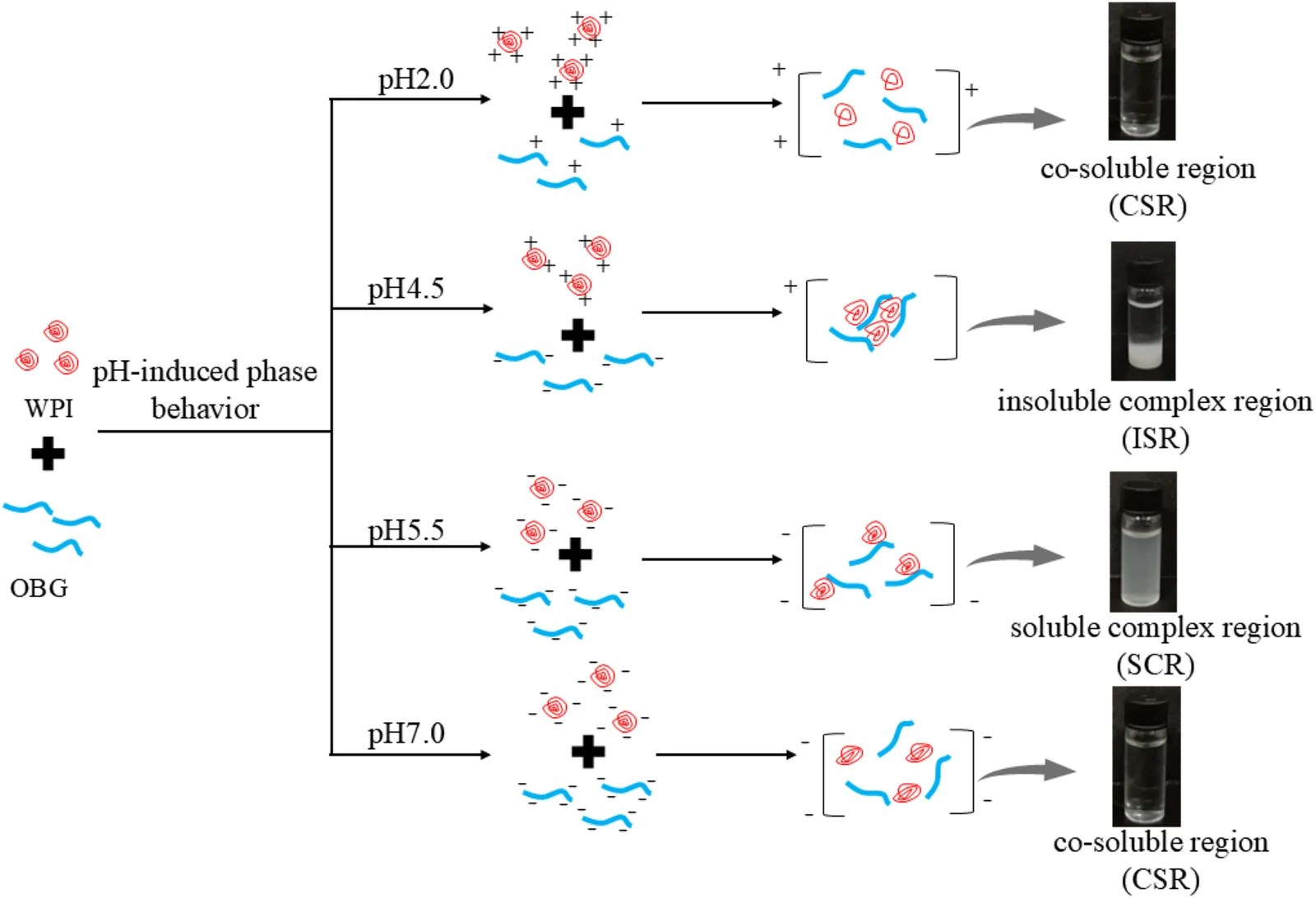
The discrepancy mechanism of structure, functionality and bioactive properties of WPI-OBG complex exposed prior to pH-induced phase transition.

Fig. 2 shows the mechanism underlying the differences in structure, functionality, and bioactive properties of WG under pH-induced phase transition. In CSRpH2.0, partial protonation imparted positive charges to OBG, while WPI also carried strong positive charges. The electrostatic repulsion between WPI and OBG maintained the mixture in a co-soluble state. The content of β-turn and random coil in WG was highest at CSRpH2.0 compared to the other phase regions. The highly acidic condition in CSRpH2.0 facilitated the disruption of intramolecular interactions within WPI molecules, promoting their conformational unfolding. In contrast, at CSRpH7.0, both WPI and OBG carried strong negative charges. The interaction between WPI and OBG remained electrostatically repulsive. In SCRpH5.5, the absolute zeta potential values of both WPI and OBG decreased, leading to weakened electrostatic repulsion between them and facilitating the formation of soluble complexes. In ISRpH4.5, the OBG carried negative charges, while WPI remained positively charged. The interaction between WPI and OBG was electrostatic attraction, which promoted the formation of WPI-OBG complexes. Science the zeta potential of WG in this phase region was close to 0, aggregation of WPI-OBG complexes occurred, accompanied by precipitation.

### 3.3 TEM analysis

**Fig. 3:**
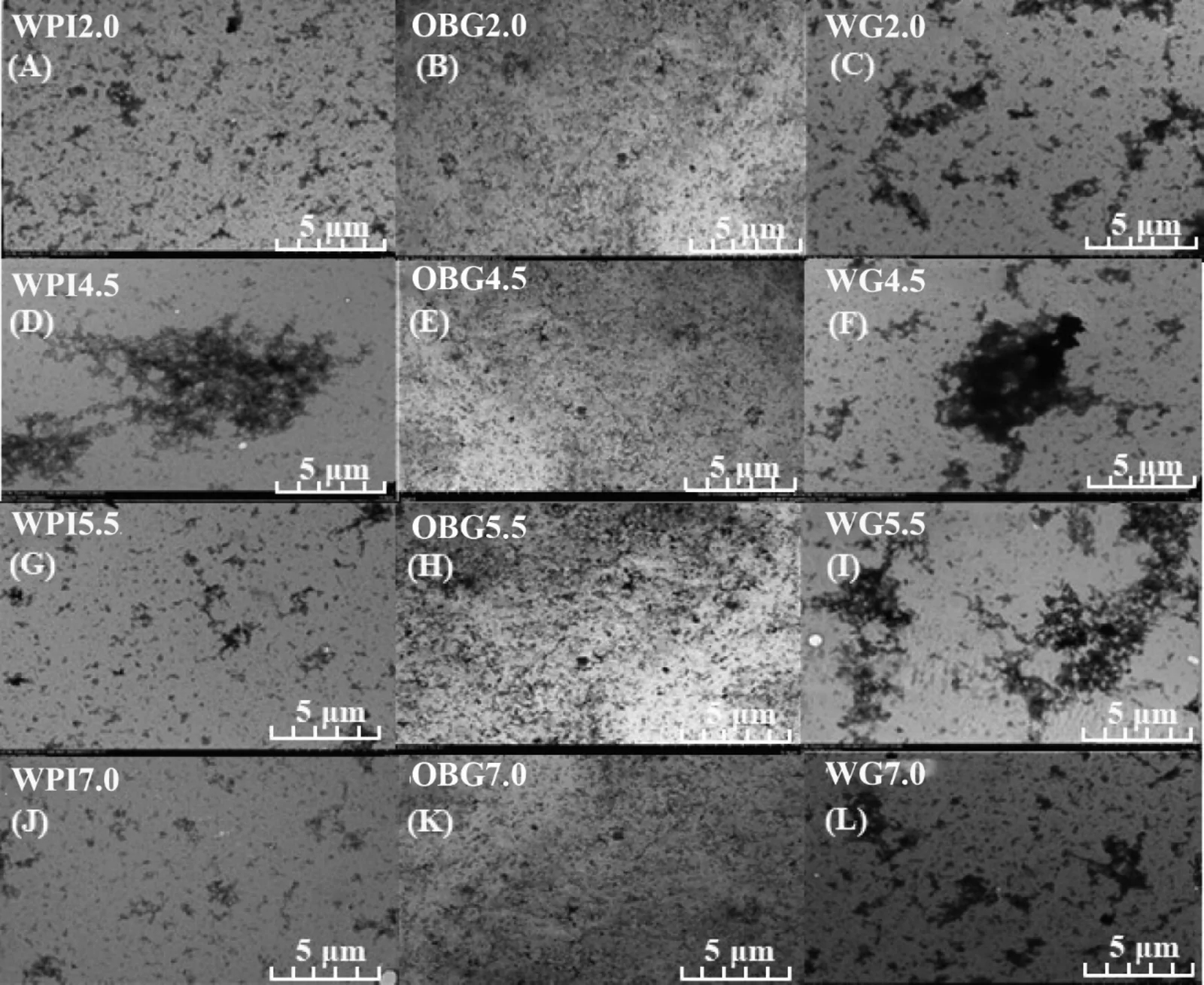
Transmission electron microscope images of WPI, OBG and WG in different phase regions.

The microstructure of WPI, OBG, and WG in different phase regions was visualized by TEM in Fig. 3. WPI showed black agglomerated particle (Fig. 3A, D, G and J). In CSRpH2.0 and CSRpH7.0, WPI exhibited relatively uniform structures (Fig. 3A and 3 J). WPI formed the largest aggregation structures in ISRpH4.5 compared with other phase regions, due to the pH being close to its pI (Fig. 3D). The aggregation structures of WPI were also found in SCRpH5.5 but its aggregates were more dispersed than those in ISRpH4.5. A similar aggregation phenomenon was also found in soybean protein when the pH was close to pI (Feng et al., 2024). The structure of OBG was a cloud-like black structure and showed no difference in different phase regions (Fig. 3B, E, H and K). Compared with WPI, the WG showed larger black aggregates in the same phase region. WG in ISRpH4.5 showed black opaque aggregates (Fig. 3F). The zeta potential of WG in ISRpH4.5 was close to 0 (shown in Fig. 2C), and WPI-OBG complex formed in WG in this phase region were prone to aggregation. The WG was black flocculent substance in SCRpH5.5 (Fig. 3I). The distribution of WG in SCRpH5.5 was more uniform without large aggregates compared to WG in ISRpH4.5. In addition, in CSRpH2.0 and CSRpH7.0, the aggregated structure of WG was less pronounced than those in SCRpH5.5 and ISRpH4.5 because the electrostatic repulsion was the main interaction force between WPI and OBG, thus reducing the formation of WPI-OBG complex aggregates (Fig. 3C and L). The particle size of WG in different phase regions decreased in the following order: ISRpH4.5 > SCRpH5.5 > CSRpH2.0 ≈ CSRpH7.0, which was consistent with the results of particle size (shown in Fig. 1B).

### 3.4 Fluorescence spectroscopy

**Fig. 4:**
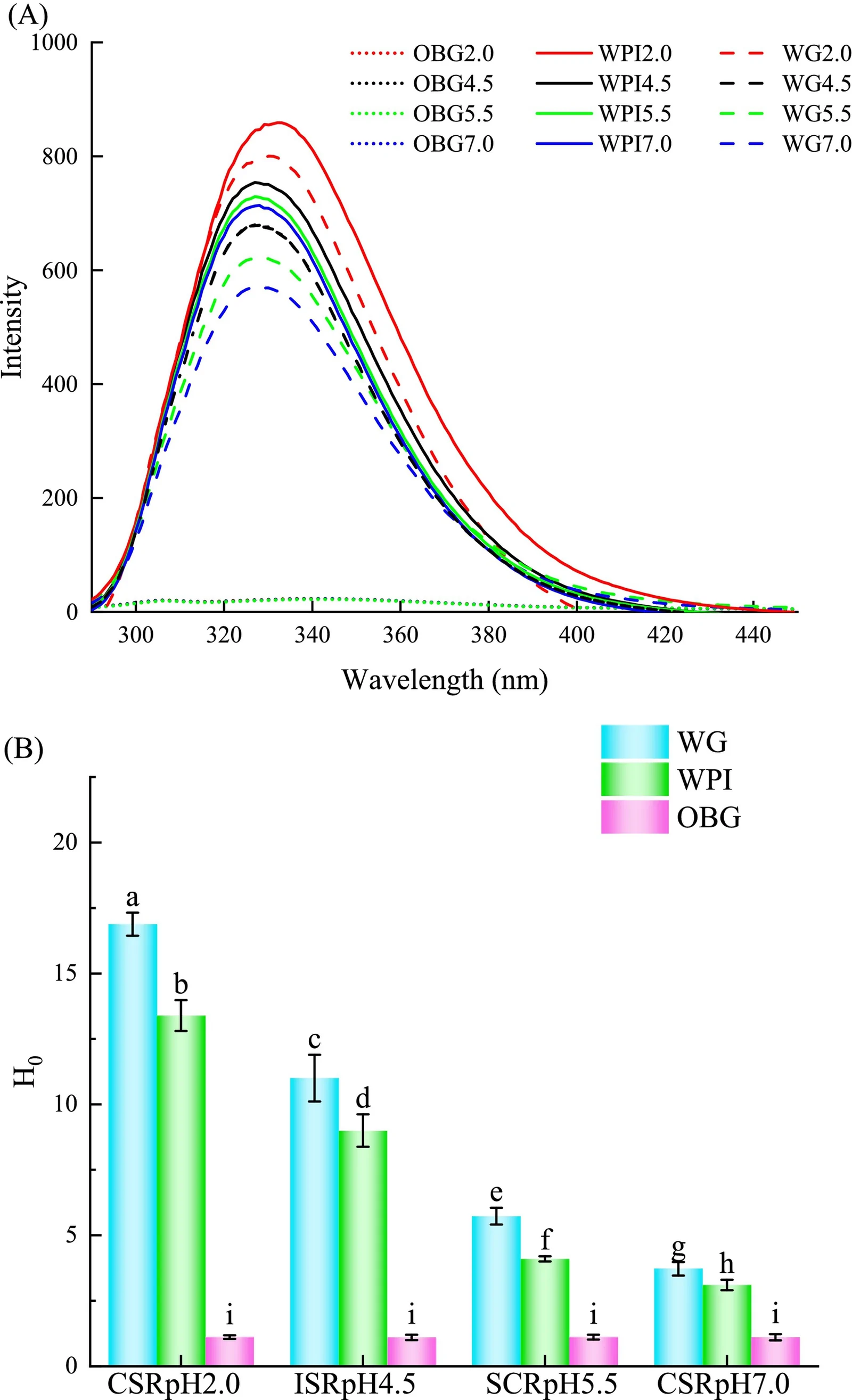
The fluorescence spectroscopy (A) and surface hydrophobicity (H_0_) (B) of WPI, OBG and WG at different phase regions.

Fluorescence spectroscopy is widely used to study changes in protein conformation and interactions between proteins and other molecules (Hinderink et al., 2021). Fig. 4A shows the fluorescence spectroscopy of WPI, OBG and WG. The fluorescence intensity of WPI in CSRpH2.0, ISRpH4.5 and SCRpH5.5 increased by 25.12%, 8.58% and 3.27%, respectively, compared with that in CSRpH7.0. There was no significant difference in fluorescence intensity of OBG between different phase regions (*P* *>* *0.05*). For WG, its fluorescence intensity among different phase regions was arranged in descending order of CSRpH2.0 > ISRpH4.5 > SCRpH5.5 > CSRpH7.0. It might be attributed to the pH differences among the different phase regions. Acidic conditions promoted the loose structure of protein (Liu et al., 2022), exposing more fluorescent groups (Try) and causing an increase in the fluorescence intensity of WPI. The fluorescence intensity of WG was lower than that of WPI because the formation of complex masked the fluorescent groups. A similar decrease was also found in soybean soluble polysaccharide-milk protein interactions (Li et al., 2015).

### 3.5 Surface hydrophobicity

Surface hydrophobicity is used for investigating the interaction between proteins and polysaccharides and is also related to the functional properties of proteins, such as emulsification and foaming properties (Chen et al., 2019). Fig. 4B displays surface hydrophobicity of WPI, OBG and WG in different phase regions. The surface hydrophobicity of WPI in CSRpH2.0 was the highest, following by ISRpH4.5, SCRpH5.5 and in CSRpH7.0. Besides, there were no significant differences in the surface hydrophobicity of OBG between different phase regions (*P* *>* *0.05*). The surface hydrophobicity of WG was notably raised after adding OBG under the same phase condition (*P* < 0.05). The structure of WG was more flexible and disordered than that of WPI (shown in Fig. 6B), which exposed a greater number of hydrophobic groups. It has also been found that oat β-glucan increased the surface hydrophobicity of heat-induced WPI-pea protein aggregates (Zou et al., 2026). Moreover, the surface hydrophobicity of WG between various phase regions was descended following order of CSRpH2.0 > ISRpH4.5 > SCRpH5.5 > CSRpH7.0. The acidic condition induced the unfolding of WPI, leading to the exposure of hydrophobic groups.

### 3.6 Three-dimensional fluorescence spectroscopy

**Fig. 5:**
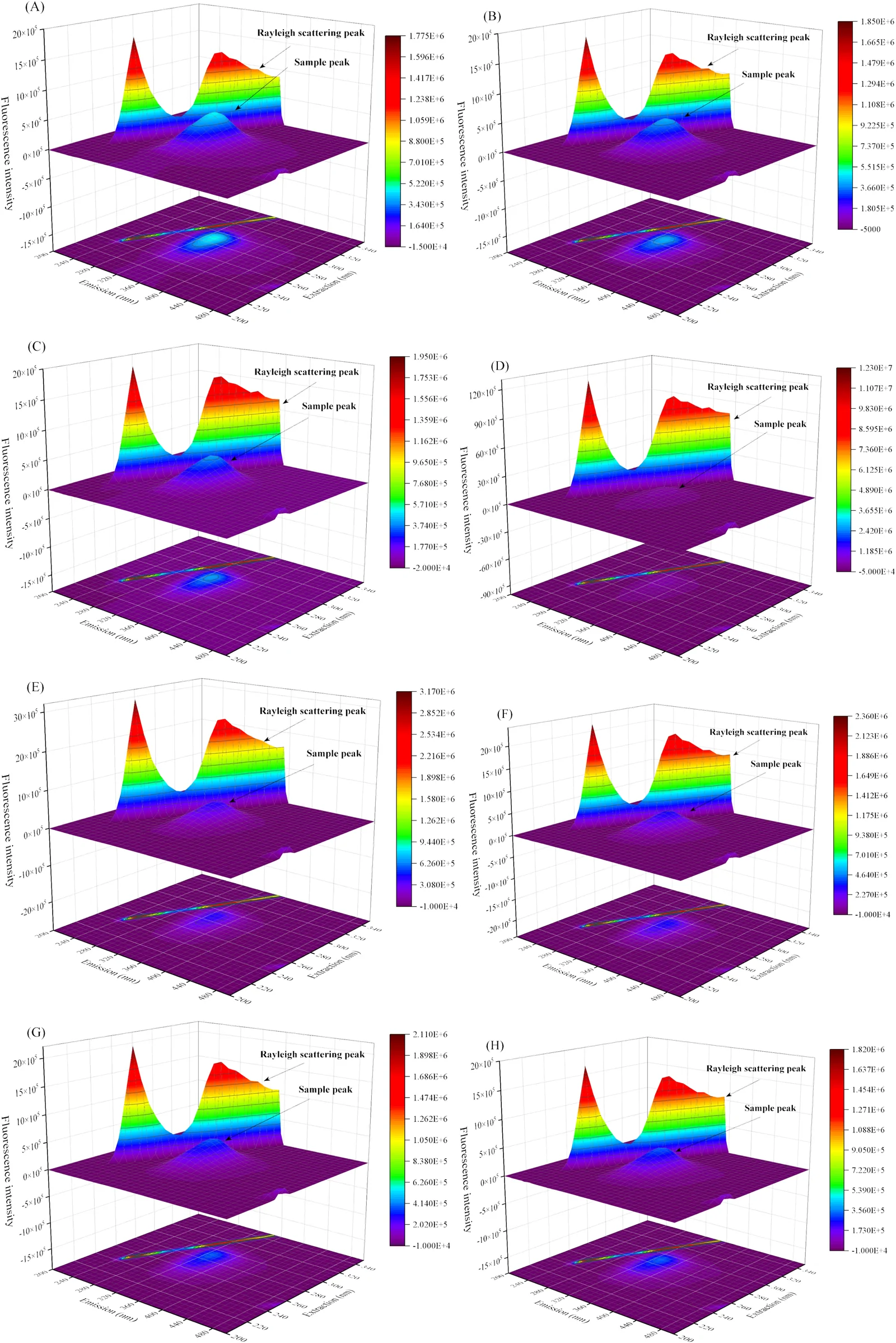
Three-dimensional synchronous fluorescence spectrum of WPI and WG at different phase regions. (A) WPI2.0, (B) WG2.0, (C) WPI4.5, (D) WG4.5, (E) WPI5.5, (F) WG5.5, (G) WPI7.0, (H) WG7.0.

Three-dimensional fluorescence spectroscopy is applied for obtaining detailed information of protein conformation and microenvironmental changes within the system (Kong et al., 2020). Since the fluorescence intensity of OBG was very low and no characteristic peaks were observed in its three-dimensional fluorescence spectra, only WPI and WG in different phase regions were analyzed. As shown in Fig. 5, the more prominent “ridges” correspond to the “pencil-type” peak in the intensity contour plot, where λ_ex_ = λ_em_ (i.e., Δλ = 0), which is known as the Rayleigh scattering peak (Luo et al., 2006). The single peak on the right side represented the three-dimensional fluorescence peak of the sample, with the lighter blue color at the top indicating higher fluorescence intensity. Furthermore, the top color of fluorescence peak was dark after the formation of WPI-OBG complex. It might be related to the interaction between WPI and OBG, which quenched the intrinsic fluorescence. The fluorescence intensity was also reduced after the formation of xanthan gum-lysozyme complex (Zhu et al., 2018). In addition, the peak colors of WG in different phase regions, from darkest to lightest, were as follows: CSRpH2.0 > CSRpH7.0 > SCRpH5.5 > ISRpH4.5. This was affected by the aggregation of WPI-OBG complex. When WG was in ISRpH4.5, the detectable chromophores were reduced due to the tight combination between WPI-OBG complex. Moreover, less aggregation occurred among complexes in SCRpH5.5 than that in ISRpH4.5, thus masking a few chromophores of WPI. In CSRpH2.0 and CSRpH7.0, there were less complex formation due to the repulsive interaction, thereby not hiding the chromophores.

### 3.7 FTIR analysis

**Fig. 6:**
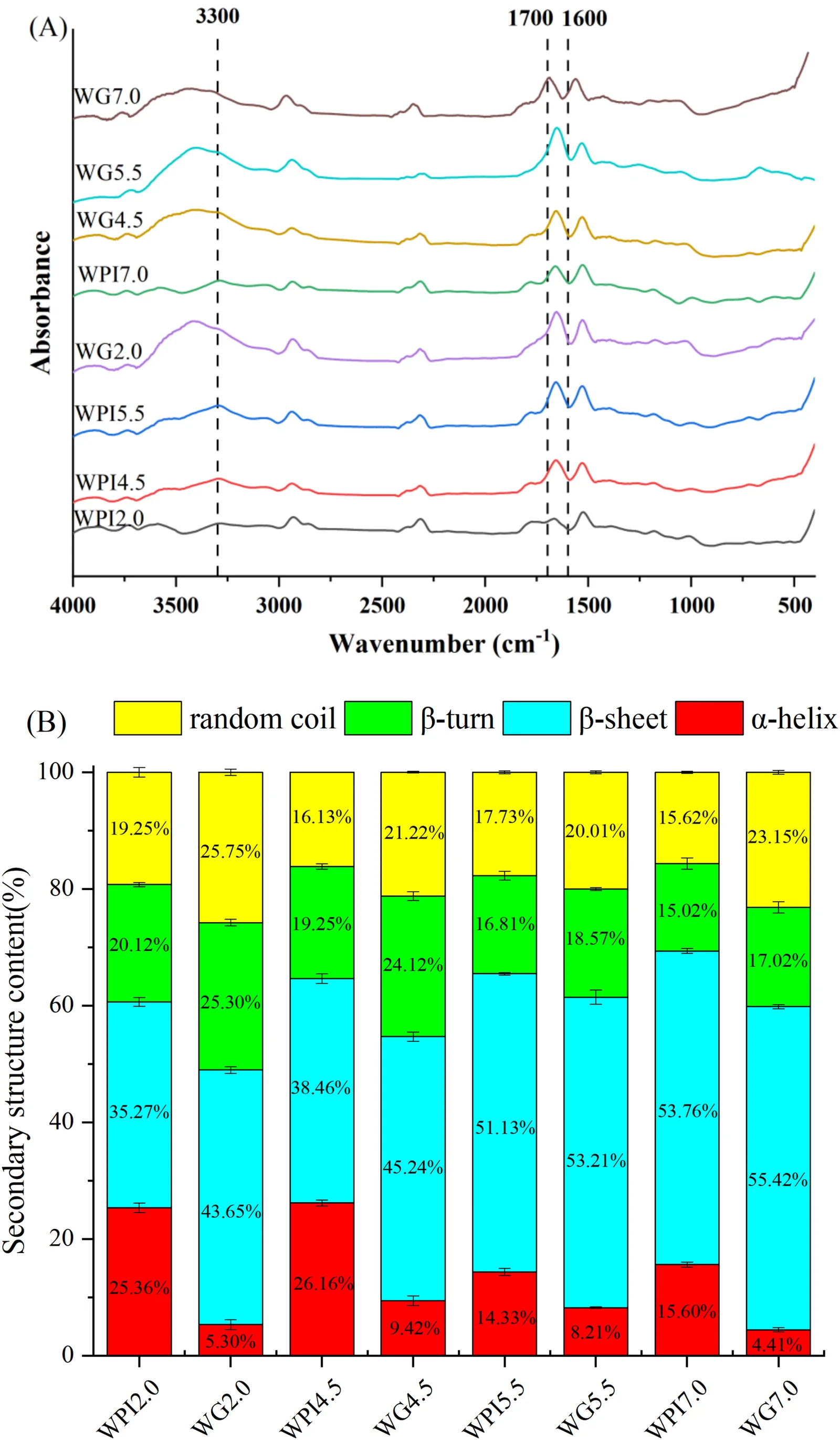
Analysis of FTIR spectra of WPI and WG. The peak shifts of WPI and WG in the region from 400 to 4000 cm^−1^. The Secondary structures composition of WPI and WG (B).

FTIR spectroscopy analysis is used to characterize the changes in secondary structure protein of proteins (Sandt, 2024). Fig. 6 shows the absorption peak of WPI, OBG, and WG in different phase regions. As shown in Fig. 6A, the absorbance of WG was increased compared with WPI. It indicated that the secondary structure of WPI was changed after the formation of WPI-OBG complex. In addition, the peaks of WG were moved to low wavelengths compared with WPI at 1700–1600 cm^−1^. It might be associated with electrostatic interactions between WPI and OBG (Li & Chen, 2022). Moreover, the peaks of WG were moved to high wavelengths compared with WPI in 3400–3200 cm^−1^. The blue shift indicated a weakened hydrogen-bonding network in WG, which was attributed to the competitive binding of water molecules by OBG, thereby disrupting the interactions between WPI and water molecules. It was also found that the hydrogen bonding between model miniproteins and water molecules was weakened due to the presence of glucose (Olgenblum et al., 2023).

Quantitative analysis of secondary structure was further carried out by deconvolution and multi-band fitting methods using peakfit 4 software in Fig. 5B. The α-helix, β-sheet, β-turn and random coil are correlated with the flexibility of the protein (Li & Arakawa, 2019). Compared with WPI, WG had more disordered structures (β-turn and random coil) in the same phase region. The structure of WPI became looser after the formation of WPI-OBG complex. For three phase regions, the WG in CSRpH2.0 had the most disordered structures, followed by ISRpH4.5, SCRpH5.5 and CSRpH7.0. Mainly, acidic conditions promoted WPI unfolding in CSRpH2.0, thus increasing the disordered structures of WG.

### 3.8 Foaming properties

**Fig. 7:**
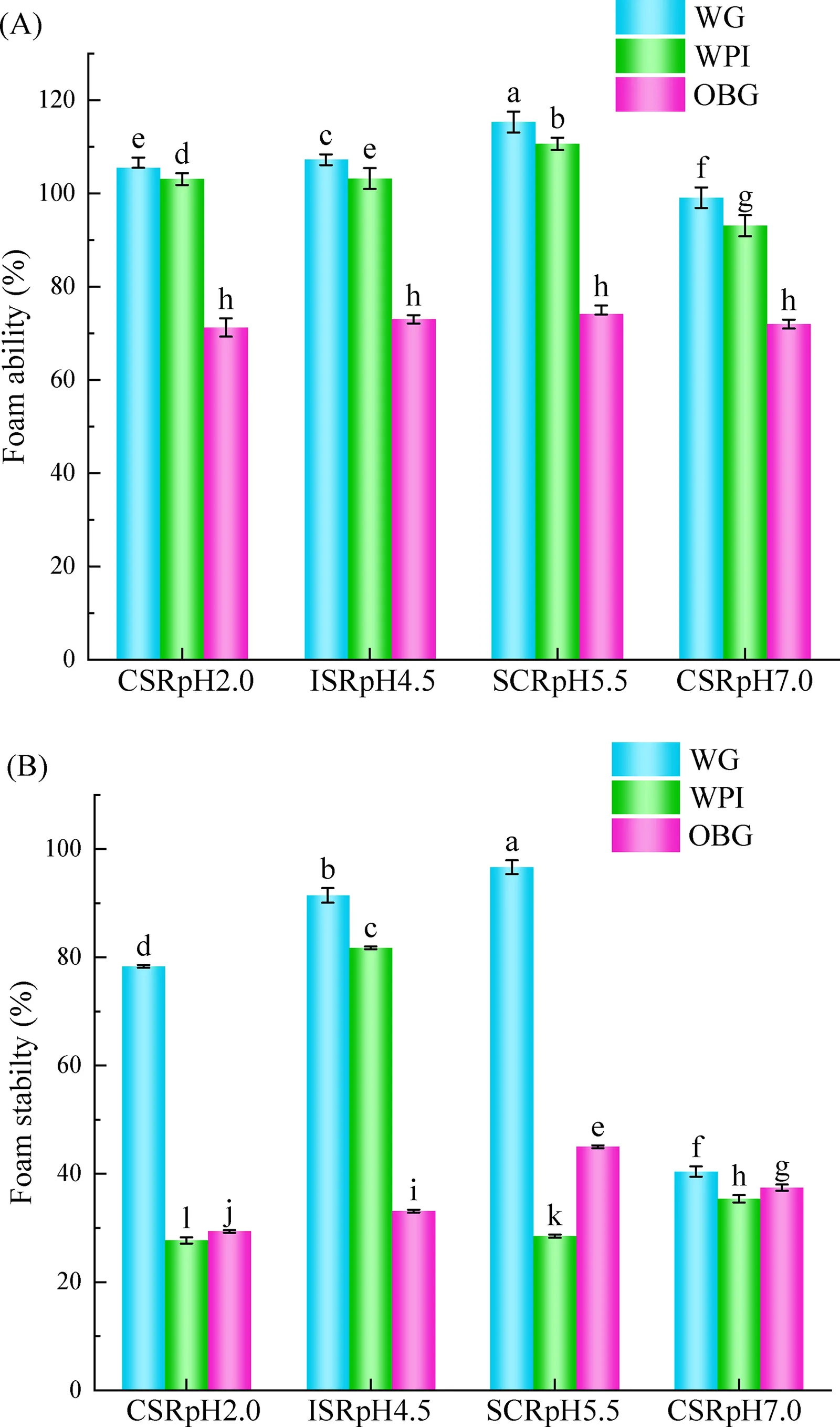

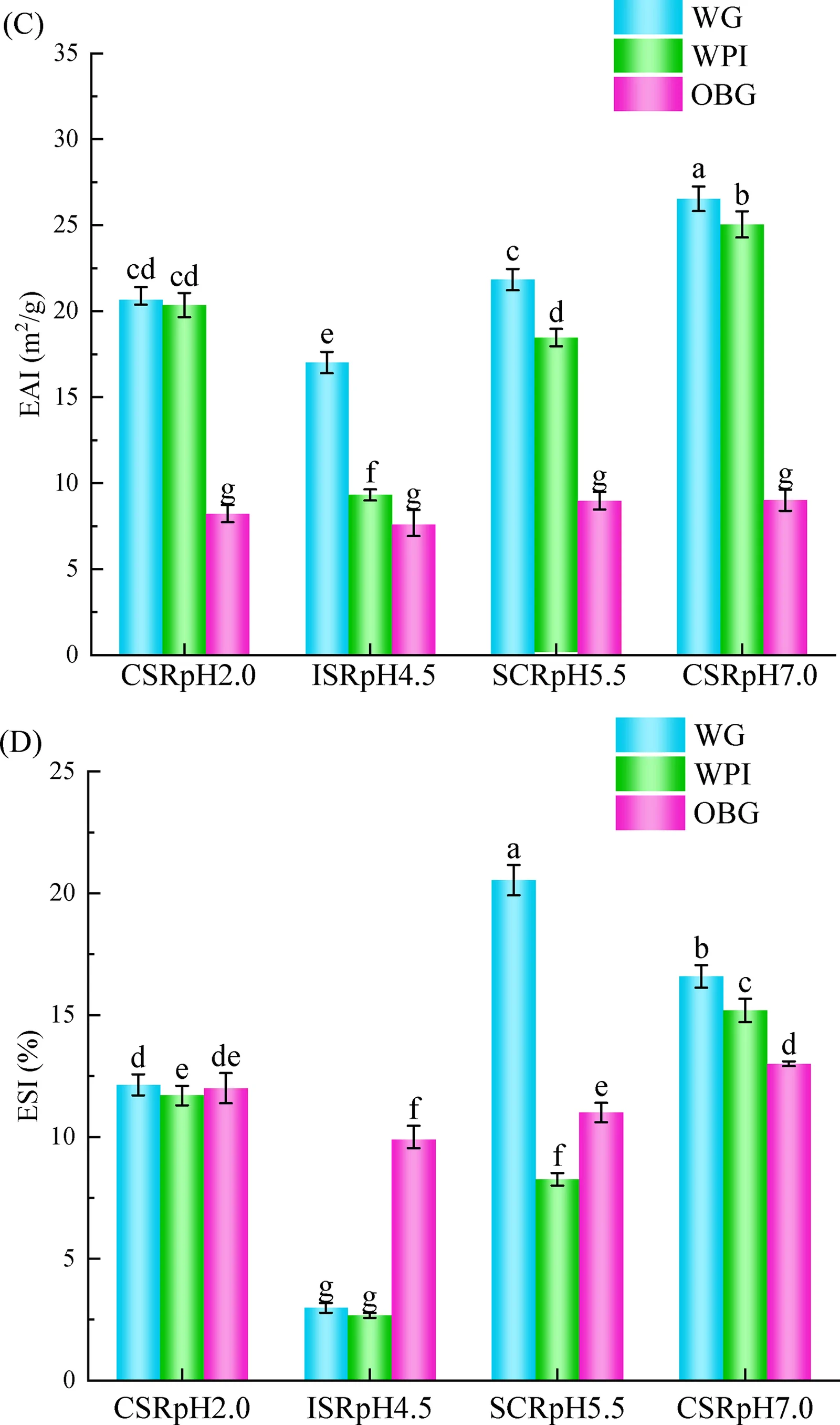
Foam ability (A), foam stability (B), emulsifying activity (C) and emulsifying stability (D) of WPI, OBG and WG in different phase regions.

The foaming properties are important for developing high quality of aerated foods (Han et al., 2023). Fig. 7A and B show the FA and FS of WPI, OBG, and WG at different phase regions. The FA and FS of WG were significantly higher than those of WPI or OBG at the same phase region (*P* < 0.05). Firstly, the conformation of WG was more flexible than that of WPI (Fig. 6B), enabling more hydrophobic sites to be exposed through rapid structural rearrangement during foam formation and thereby facilitating its fast adsorption at the A/W interface. Oduse et al. (2017) also concluded that FA of whey protein was enhanced by non-covalent binding to pectin. Moreover, the introduction of polysaccharide enhanced the viscosity of WG, which reduced diffusion rate of aqueous phase, thus effectively slowing foam drainage and coarsening (Moll et al., 2020).

In different phase regions, the foaming properties of WG in ISRpH4.5 were poorer compared with SCRpH5.5. The particle size of WG in ISRpH4.5 was bigger than that of WG in SCRpH5.5 (shown in Fig. 1B), resulting in a lower diffusion rate toward the A/W interface, which was unfavorable for interfacial adsorption at the A/W interface and stabilization of foams. It was also reported that WPI-alginate soluble complex had better foaming properties than insoluble complex (Xu et al., 2020). Moreover, FA and FS of WG in CSRpH2.0 were significantly higher than that of WG in CSRpH7.0. More hydrophobic groups in WG were exposed in CSRpH2.0 compared to CSRpH7.0 (shown in Fig. 4B), which strengthened hydrophobic interactions between WG and A/W interface, thereby accelerating its adsorption at A/W interface. Further, the increased hydrophobic groups of WG enhanced its interfacial anchoring, thus increasing its interfacial adsorption stability.

### 3.9 Emulsifying properties

Fig. 7C and D present the emulsifying properties of WPI, OBG, and WG in three phase regions. In Fig. 7C, the emulsifying activity index (EAI) of WG was higher than WPI or OBG in the same phase region. The surface hydrophobicity of WG was higher than WPI (shown in Fig. 4B), which promoting hydrophobic interaction between WG and oil droplets, thus strengthening its affinity for the O/W interface and facilitating its rapid and stable adsorption. It was also reported that emulsifying properties of inulin-protein complex were enhanced compared with single protein (Liu et al., 2016). Moreover, WG in SCRpH5.5 had more excellent EAI than that in ISRpH4.5. WG in SCRpH5.5 exhibited smaller particle size than WG in ISRpH4.5 (shown in Fig. 1B), causing the increase of specific surface area and diffusion rate, thus facilitating its rapid adsorption at the O/W interface.

Fig. 7D shows the ESI of WPI, OBG and WG at different phase regions. The ESI of WG in SCRpH5.5 was higher than that of WG in CSRpH2.0 and CSRpH7.0. It was also found that the ESI of α-lactalbumin-chitosan soluble complex was higher than that of α-lactalbumin-chitosan in co-soluble state (Liu et al., 2020). The formation of large soluble complex aggregation (shown in Fig. 1B) in SCRpH5.5 contributed to an increase in emulsion viscosity, which effectively restricted oil droplets mobility and coalescence, thereby improving emulsion stability. The ESI of WG in ISRpH4.5 was the lowest compared with that in other phase regions. The absolute value of the zeta potential of WG in ISRpH4.5 was the lowest (shown in Fig. 1C), resulting in insufficient electrostatic repulsion at the O/W interface to maintain the stability of the emulsion. It was also reported that insoluble protein-polysaccharide complex played a deleterious role for emulsions stability (Laplante et al., 2006).

### 3.10 Antioxidant properties

**Fig. 8:**
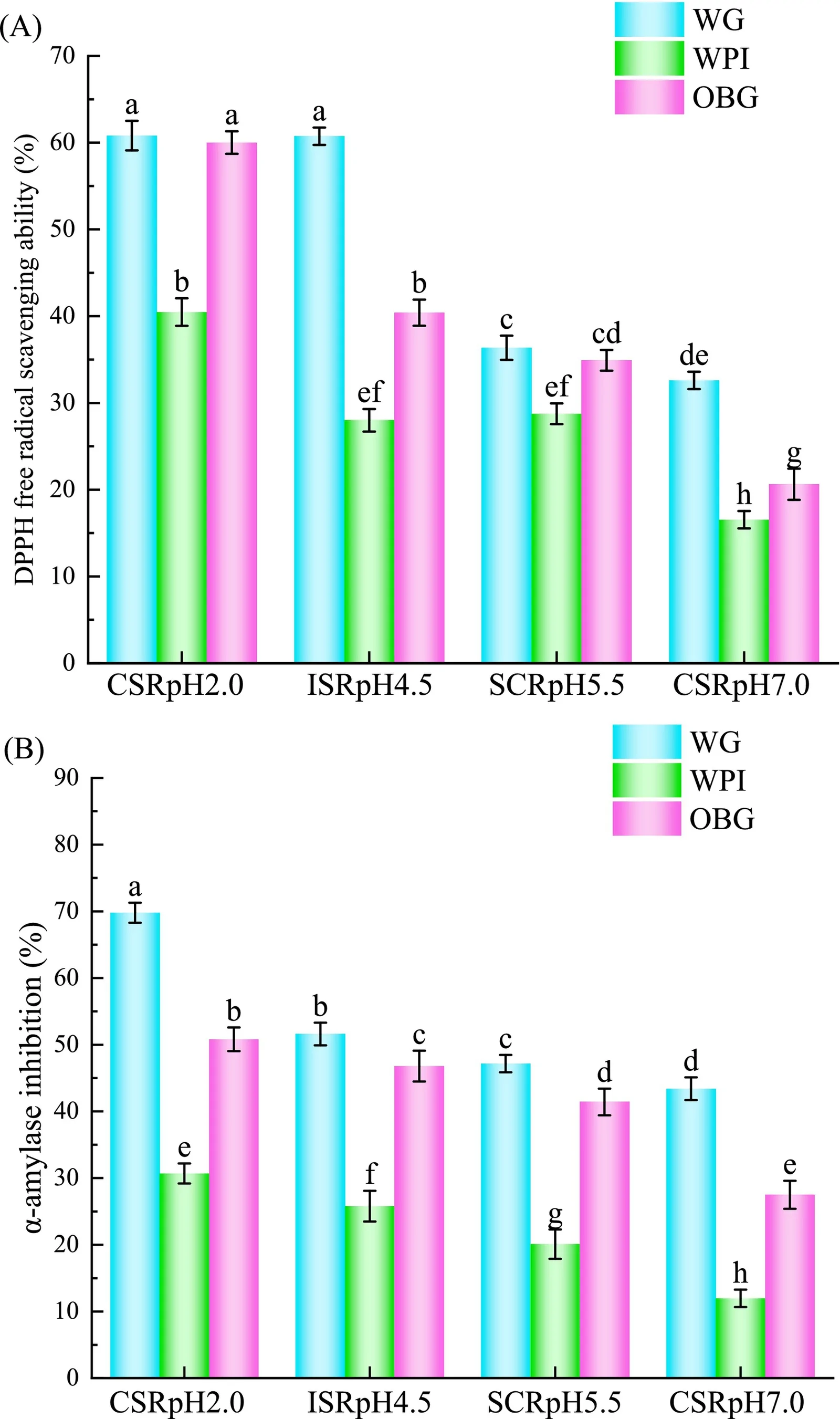

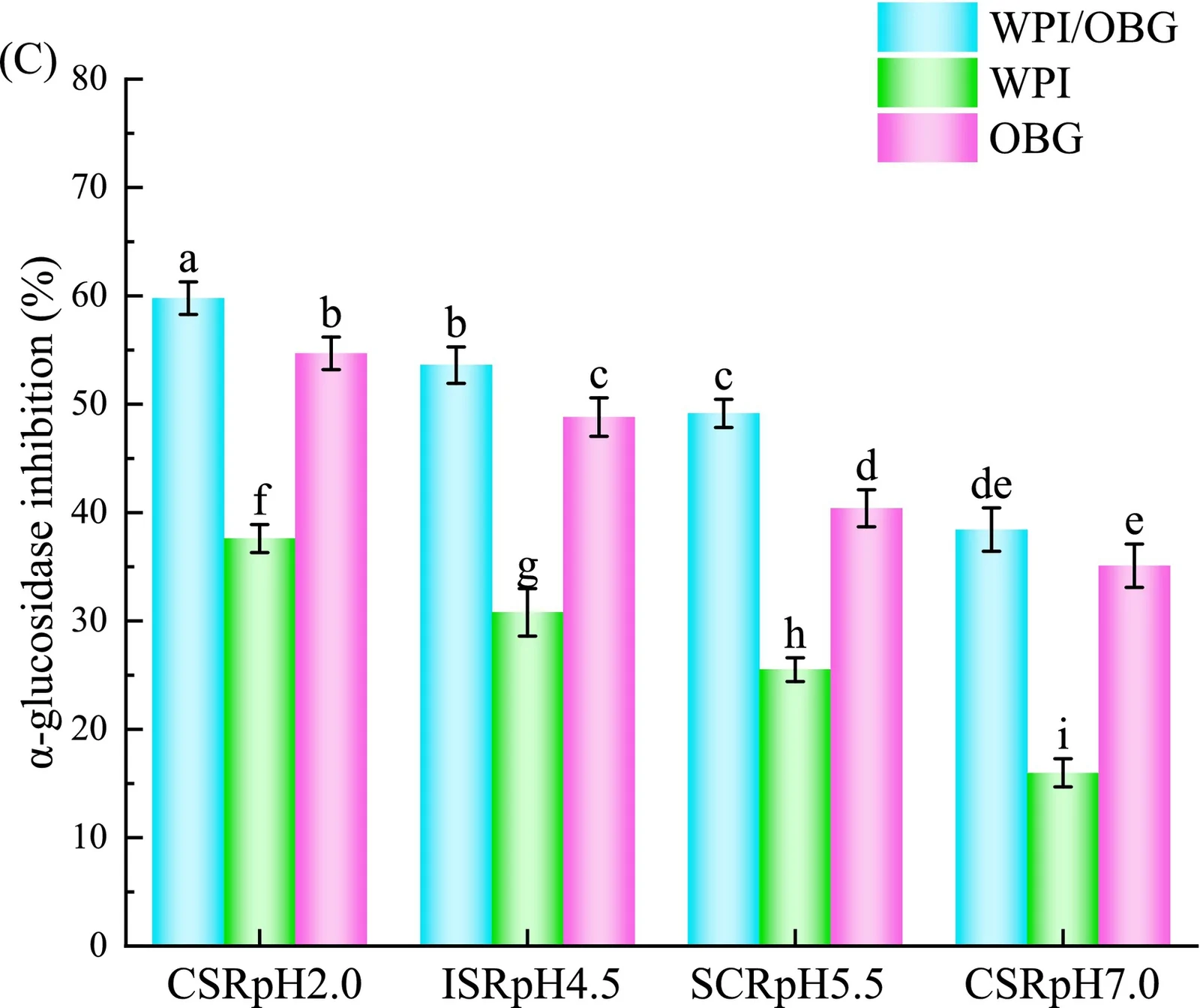
The DPPH free radical scavenging capacity (A) and inhibitory ability of α-Amylase (B), α-glucosidase (C) of WPI, OBG and WG in different phase regions.

DPPH free radical scavenging capacity is commonly used to assess the antioxidant capacity (Phillips et al., 2011). Fig. 8A displays the DPPH free radical scavenging capacity of WPI, OBG, and WG in different phase regions. WG had significantly higher DPPH free radical scavenging capacity in CSRpH2.0 than that in SCRpH5.5 and CSRpH7.0 (*P* < 0.05). In CSRpH2.0, most aromatic amino side chains (tryptophan, tyrosine, and phenylalanine) of WG were exposed, compared other phase regions (shown in Fig. 4). These amino acid residues could readily donate hydrogen and inactivate the DPPH radicals (Elias et al., 2008). Moreover, WG in ISRpH4.5 also had a significantly higher DPPH free radical scavenging capacity than that in SCRpH5.5 and CSRpH7.0 (*P* < 0.05).

### 3.11 Inhibitory ability of α-amylase and α-glucosidase

Fig. 8B and C show the inhibitory ability of α-amylase(B) and α-glucosidase(C) of WPI, OBG and WG in different phase regions. WG had significantly stronger inhibitory abilities against α-amylase and α-glucosidase than WPI or OBG in the same phase regions (*P* < 0.05). WG also had higher viscosity than WPI or OBG (Hu et al., 2025a). The high viscosity of WG prevented enzyme diffusion and reduced contact between enzyme and substrate. Furthermore, WG in CSRpH2.0 had the maximum value of α-amylase/α-glucosidase inhibitory ability, closely followed by ISRpH4.5, SCRpH5.5 and CSRpH7.0. It has been found that hydrophobic amino acid residues in proteins, such as leucine, glycine, proline, and phenylalanine, can bind to the active sites of α-amylase and α-glucosidase (Baba et al., 2021), thus inhibiting the absorption of starch or glucose. WG in CSRpH2.0 exposed more hydrophobic amino acid residues (shown in Fig. 4B) and might interacted more strongly with α-amylase and α-glucosidase than WG in other phase regions.

### 3.12 PCA and heat map analysis

**Fig. 9:**
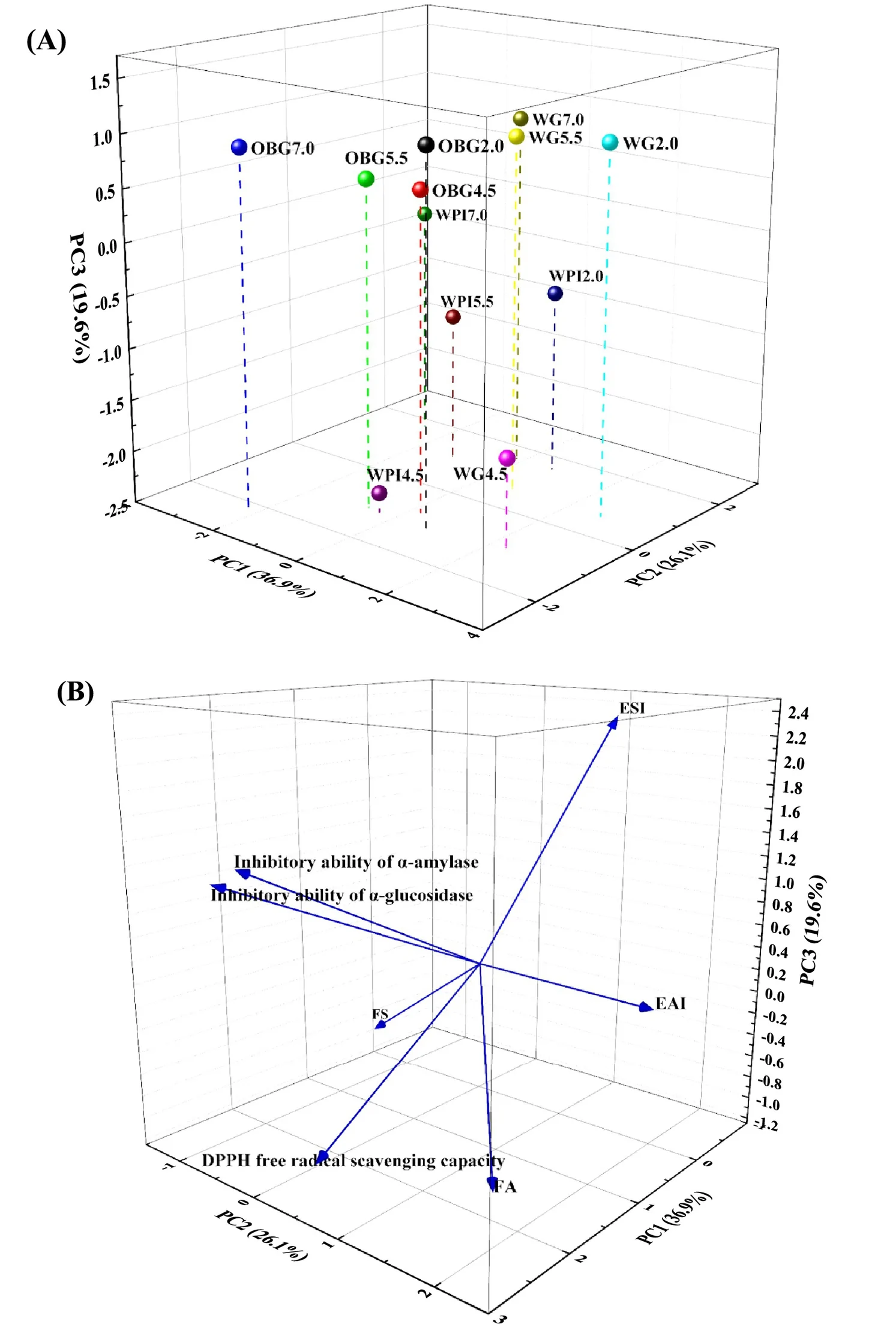

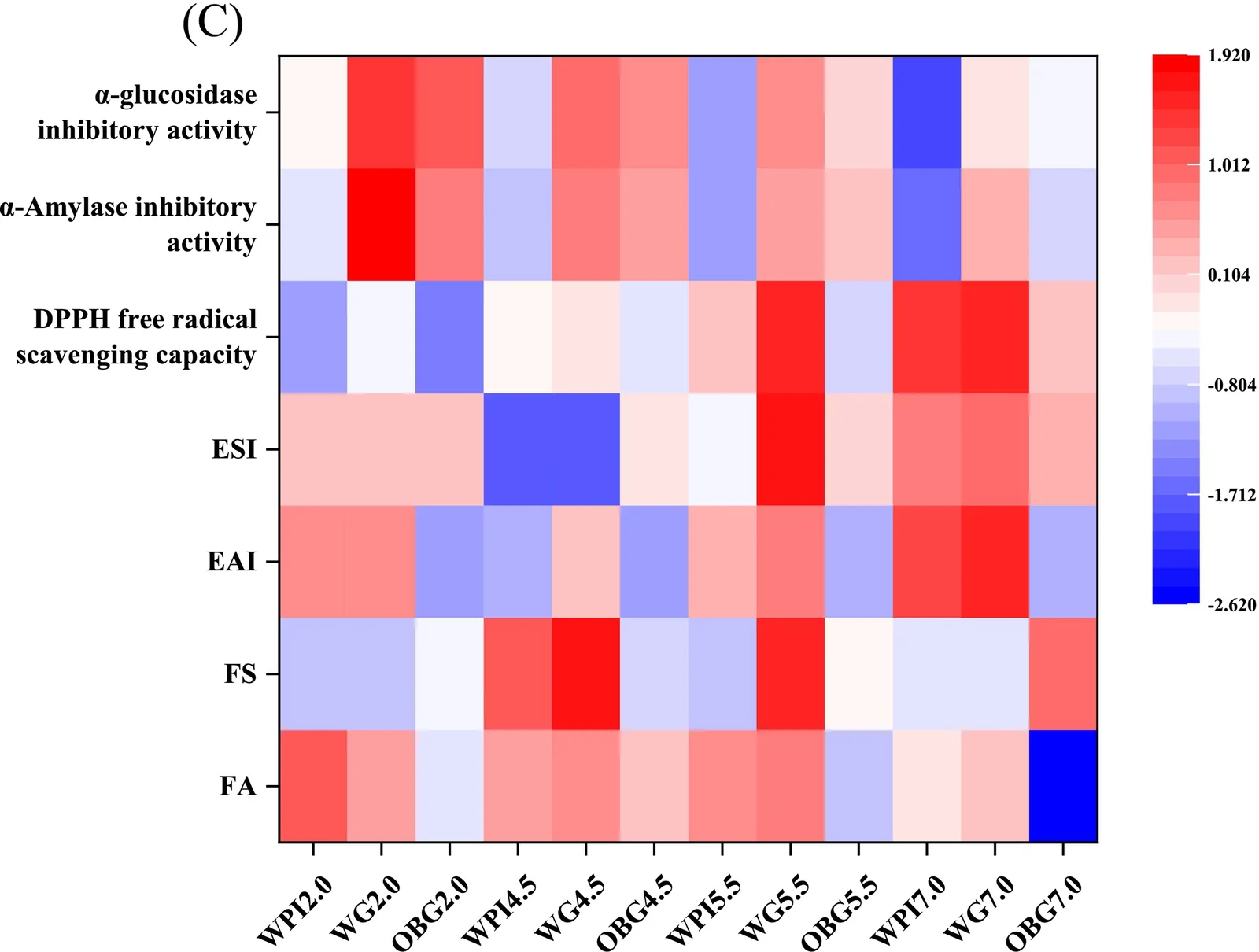
Principal component analysis [load diagram (A) and score chart (B)] and heatmap analysis (C) of WPI, OBG and WG in different phase regions.

PCA was used to transform and downscale the data for each indicator of WG in three phase regions. In Fig. 9A and B, PC1, PC2 and PC3 reflected 36.9%, 26.1% and 19.6% of the total information, respectively. WG in CSRpH2.0 and SCRpH5.5 was located in the first quadrant. WG in ISRpH4.5 was located in the eighth quadrant. WG in CSRpH7.0 was located in the ninth quadrant. This indicated that the functional properties and biological activities of WG differed among the phase regions. Meanwhile, WG in CSRpH2.0 had the highest comprehensive score among the different phase regions.

In order to further visually compare the functional and bioactive properties of WPI-OBG complex in three phase regions, the heat map was used to convert the data into the form of chromaticity. In Fig. 9C, compared with WPI in the same phase region, WG exhibited superior functional and bioactive properties. WG in SCRpH5.5 had the highest foaming properties and emulsion stability compared with those in other phase regions. WG in CSRpH2.0 possessed the strongest DPPH free radical scavenging capacity and inhibitory ability of α-amylase and α-glucosidase compared with those in other phase regions. Emulsifying activity index of WG in CSRpH7.0 was the highest among other phase regions. The foaming properties of WG in ISRpH4.5 was better than those in CSRpH2.0 and CSRpH7.0, but lower than those of WG in SCRpH5.5.

## 4 Conclusion

The four phase regions, including CSRpH2.0, ISRpH4.5, SCRpH5.5, and CSRpH7.0, were identified by turbidity analysis of WG at different pH values. The functionality and bioactive properties of WG were significantly higher than WPI (*P* < 0.05). Across all phase regions, WG at SCRpH5.5 displayed the highest foaming capacity, while WG at ISRpH4.5 exhibited the poorest emulsification properties, attributed to its largest particle size. Based on PCA analysis, WG at CSRpH2.0 achieved the most favorable comprehensive scores for functionality and in vitro bioactivity among all phase regions. These findings provide a fundamental basis for designing the protein-polysaccharide functional ingredients with targeted properties. However, the advantages of WG from different phase regions remain to be validated in practical application within diverse food matrices. In further work, the application of protein-polysaccharide non-covalent complexes as stabilizer or functional ingredient would be studied to acquire food with excellent properties.

## CRediT authorship contribution statement

**Heyang Xu:** Writing – original draft, Formal analysis, Data curation. **Zhishen Mu:** Writing – original draft, Funding acquisition, Conceptualization. **Han Wang:** Visualization, Supervision, Project administration. **Xin Wen:** Writing – original draft, Methodology, Investigation. **Jialun Hu:** Writing – original draft, Software, Project administration. **Zhanmei Jiang:** Writing – review & editing, Resources, Funding acquisition. **Munkh-Amgalan Gantumur:** Writing – review & editing, Validation, Conceptualization.

## Declaration of competing interest

The authors declare that they have no known competing financial interests or personal relationships that could have appeared to influence the work reported in this paper.

## Data Availability

The authors do not have permission to share data.
